# Comprehensive Ontology of Fibroproliferative Diseases: Protocol for a Semantic Technology Study

**DOI:** 10.2196/48645

**Published:** 2023-08-11

**Authors:** Marcin Golec, Maulik Kamdar, Sandra Barteit

**Affiliations:** 1 Heidelberg Institute of Global Health Faculty of Medicine and University Hospital Heidelberg University Heidelberg Germany; 2 Center for Advanced Clinical Solutions Optum Health Eden Prairie, MN United States

**Keywords:** fibroproliferative disease, fibrosis, fibrotic disease, ontology, OWL, semantic technology, Web Ontology Language

## Abstract

**Background:**

Fibroproliferative or fibrotic diseases (FDs), which represent a significant proportion of age-related pathologies and account for over 40% of mortality in developed nations, are often underrepresented in focused research. Typically, these conditions are studied individually, such as chronic obstructive pulmonary disease or idiopathic pulmonary fibrosis (IPF), rather than as a collective entity, thereby limiting the holistic understanding and development of effective treatments. To address this, we propose creating and publicizing a comprehensive fibroproliferative disease ontology (FDO) to unify the understanding of FDs.

**Objective:**

This paper aims to delineate the study protocol for the creation of the FDO, foster transparency and high quality standards during its development, and subsequently promote its use once it becomes publicly available.

**Methods:**

We aim to establish an ontology encapsulating the broad spectrum of FDs, constructed in the Web Ontology Language format using the Protégé ontology editor, adhering to ontology development life cycle principles. The modeling process will leverage Protégé in accordance with a methodologically defined process, involving targeted scoping reviews of MEDLINE and PubMed information, expert knowledge, and an ontology development process. A hybrid top-down and bottom-up strategy will guide the identification of core concepts and relations, conducted by a team of domain experts based on systematic iterations of scientific literature reviews.

**Results:**

The result will be an exhaustive FDO accommodating a wide variety of crucial biomedical concepts, augmented with synonyms, definitions, and references. The FDO aims to encapsulate diverse perspectives on the FD domain, including those of clinicians, health informaticians, medical researchers, and public health experts.

**Conclusions:**

The FDO is expected to stimulate broader and more in-depth FD research by enabling reasoning, inference, and the identification of relationships between concepts for application in multiple contexts, such as developing specialized software, fostering research communities, and enhancing domain comprehension. A common vocabulary and understanding of relationships among medical professionals could potentially expedite scientific progress and the discovery of innovative solutions. The publicly available FDO will form the foundation for future research, technological advancements, and public health initiatives.

**International Registered Report Identifier (IRRID):**

PRR1-10.2196/48645

## Introduction

### Fibroproliferative Diseases

Fibroproliferative wound healing, a process that can disrupt normal organ development and lead to increasing fibrosis and eventual organ failure, can affect nearly all organs and tissues. These fibroproliferative or fibrotic diseases (FDs) [1], including lung fibrosis, liver cirrhosis, atherosclerosis, keloids, progressive renal diseases, and systemic sclerosis, represent one of the most prevalent age-related illnesses, impacting over 800 million individuals worldwide [2]. They contribute to over 40% of mortality in developed nations [3,4], a rate that is anticipated to rise with the aging global population, signifying a growing public health concern.

Despite their significance for public health, FDs have received limited attention in both policymaking and research. While these conditions share fundamental biological processes, they are seldom approached collectively in either in vitro studies or clinical settings, constraining effective investigation of shared mechanisms and hindering the formulation of comprehensive public health policies and adequate clinical care [5].

A PubMed search conducted on November 5, 2022, yielded 140, 1674, and 369,557 records for the terms *fibroproliferative diseases*, *fibrotic diseases*, and *fibrosis*, respectively. However, these numbers are inflated by the inclusion of conditions such as *cystic fibrosis*, which is not an FD.

Significant advances have been made in recent years in understanding the molecular details of fibrosis [6,7], the causes and potential therapeutic approaches of various fibroproliferative disorders [8], and in accumulating genomic data on FDs [9]. Insights into epigenetic changes [10], noncoding RNA’s role in fibrosis [11], and the escape of fibroblasts from apoptosis [12] have also been unveiled. Promising therapeutic agents, such as dioscin for liver fibrosis and thalidomide and etanercept for lung fibrosis [13,14], are under investigation. Nevertheless, knowledge gaps remain, particularly in areas such as cardiac fibrosis [15], and efficient antifibrotic therapies are still lacking.

The vast and expanding data on FDs remain largely unsystematized and scattered, limiting our understanding of fibrosis mechanisms and hampering the development of comprehensive therapeutic strategies to target fibroproliferative processes.

### Ontology on Fibroproliferative or Fibrotic Diseases

In the realm of information technology, an ontology formally defines entities, classes, and relations, providing a structured representation of a knowledge domain [16,17]. In the context of FDs, this domain extends beyond disease nomenclature to encompass hierarchical structures, relationships between diseases, and a host of associated biomedical concepts, such as wound healing pathologies, epithelial-mesenchymal transitions, collagen deposition, involved pathways, genes, proteins, and external factors [18]. Consequently, the FD domain represents a multidimensional and multilevel ecosystem of data, information, and knowledge.

While ontologies have been developed for FD subgroups, such as the classification of fibrotic interstitial lung diseases by Ryerson et al [18] and cross-disease analyses of genetic underpinnings and implicated pathways [19-21], a comprehensive ontology encapsulating the full spectrum of fibroproliferative disorders remains lacking. This contrasts with efforts in other medical fields, such as the National Cancer Institute Thesaurus [22,23].

Clinical ontologies, like the Systematized Nomenclature of Medicine–Clinical Terms (SNOMED CT) [24-26], serve crucial roles in automating decision support tools, standardizing clinical research databases, and ensuring semantic interoperability. In the emerging field of biomedical informatics, domain ontologies enable the structured representation of concepts and entities and their relationships, forming a conceptual scaffold for domain-specific terminologies. These ontologies can be harnessed for tasks such as reasoning, inference, process identification, software enhancement, and community engagement [27-29].

Despite its potential for accelerating scientific progress and enabling novel solutions, no public FDO currently exists. Our review of the MEDLINE, PubMed, and BioPortal databases and the European Molecular Biology Laboratory-European Bioinformatics Institute (EMBL-EBI) Ontology Lookup Service confirmed this lack of a dedicated ontology for FDs [30,31].

Thus, we aim to create and disseminate an FDO as a public resource, fostering collaboration and enabling future development and refinement. This FDO targets several key objectives: sparking interest among stakeholders, facilitating public health initiatives, and supporting knowledge representation, information retrieval, data integration, health information systems, and biomedical research. We believe that a comprehensive FDO will equip researchers and clinicians to navigate the FD domain efficiently and contribute to the evolution of research and therapeutic strategies.

## Methods

In the following, we detail the methodology for the development of a comprehensive domain-specific ontology of FDs.

### Development of the Fibroproliferative Disease Ontology

The development of the fibroproliferative disease ontology (FDO) will use a top-down approach, starting with the most abstract concepts within the FD domain and then progressively refining toward specific subconcepts. This process integrates (1) findings from our comprehensive literature survey, which includes the results of scoping reviews (detailed subsequently); (2) a review of key classification systems, including the International Classification of Diseases 11th Revision (ICD-11) of the World Health Organization (WHO) SNOMED CT, and Unified Medical Language System (UMLS) Metathesaurus [32], where pertinent; and (3) the significant expertise of experienced clinicians and researchers in the fields of medicine, biomedical informatics, and public health, notably those previously involved in the large-scale Seventh Framework Program of the European Union (FP7-EU) project Resolve Chronic Inflammation and Achieve Healthy Aging by Understanding Non-regenerative Repair (RESOLVE) [1]. The ontology will be constructed using the Protégé ontology editor [33] in the Web Ontology Language (OWL) format [34].

**Figure 1:**
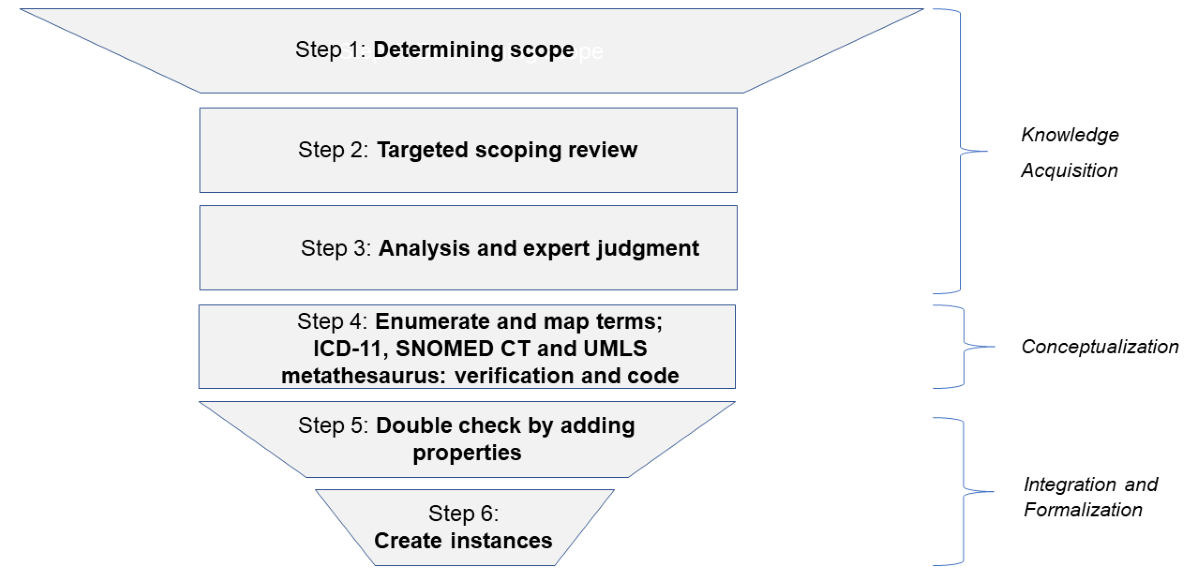
Diagram presenting steps to be applied in each phase of creating fibroproliferative or fibrotic disease ontology. Step 1, determining scope of the step based on the expert judgment analysis; step 2, targeted scoping review based on scope search defined in step 2 by experts; step 3, analysis of the scoping review results combined with experts’ domain knowledge; step 4, enumerating and mapping identified and included terms, combined with International Classification of Diseases 11th Revision verification, if relevant; step 5, double check, adding properties if relevant; step 6, translating the results into the ontology in a Protégé file by creating instances. ICD-11: International Classification of Diseases 11th Revision; SNOMED CT: Systematized Nomenclature of Medicine–Clinical Terms; UMLS: Unified Medical Language System.

**Figure 2:**
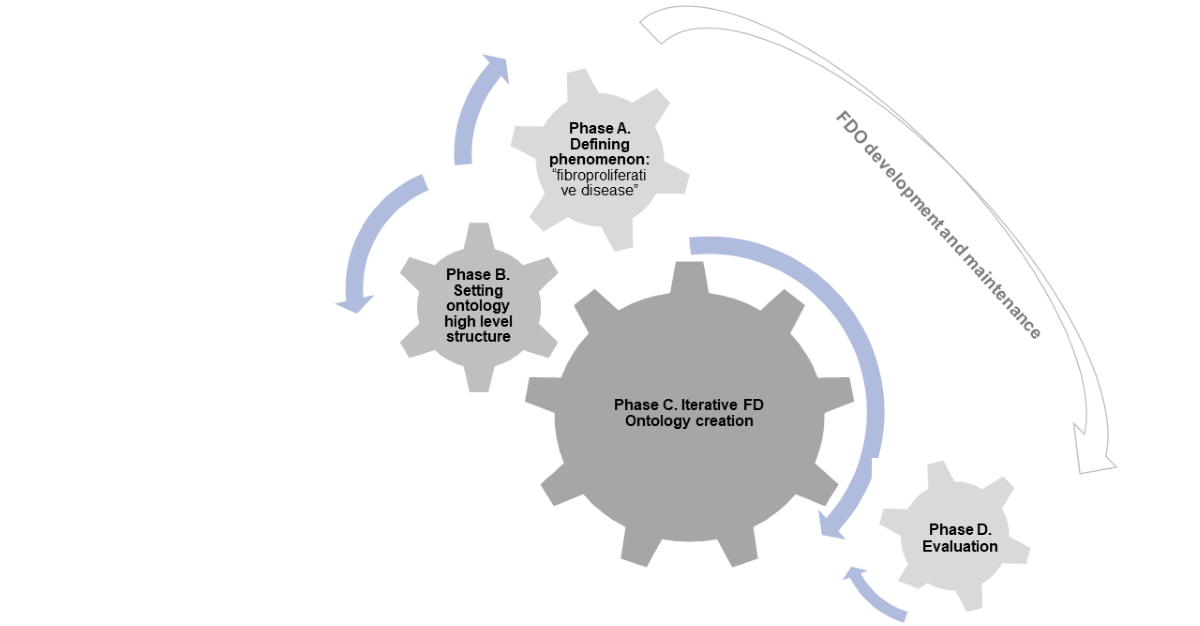
Building blocks and phases proposed for a methodological grid enabling creation of domain ontologies for complex and interdisciplinary clinical and biomedical phenomena, as exemplified by fibroproliferative or FD ontology creation. Phase A, defining a phenomenon concept, defining “fibroproliferative disease;” phase B, setting the ontology structure—determining high-level scope by expert judgment; and phase C, iterative FD ontology creation. Each of the abovementioned phases is to be implemented in a rigorous iterative row of steps. The final phase D involves an evaluation of the ontology followed by a necessary revision, if needed. Steps 1-3 represent knowledge acquisition; steps 4-5 represent conceptualization; and steps 5-6 represent integration and formalization. The chain-wheel or sprocket-wheel shape of the diagram indicates an iterative and combined approach to FD ontology building. FD: fibroproliferative or fibrotic disease.

The iterative steps for the FDO creation, illustrated in Figure 1, consist of 4 core building blocks (Figure 2) to be systematically applied:

- Building block A will see the expert team delineate the concept of FD, informed by scoping review results and their own professional insight. Here, characteristics specific to FD, including data properties like extracellular matrix (ECM) and collagen deposition and the epithelial-mesenchymal transition (EMT) phenomenon, will be specified. To capture the breadth of the FD phenomenon, the scope will span a range of major biomedical concepts, enriched with synonyms, definitions, and references as driven by scoping review findings and expert judgment. Adherence to SNOMED CT terms and granularity will be sought where feasible, with exceptions made where evidence necessitates exceeding this frame. These defining concepts will be integrated into the FDO as data and object properties unless decided otherwise.
- Building block B focuses on determining the FDO’s overall scope, envisioned to encapsulate key categories of FD. Hence, the high-level structure will pivot around body systems and organs, for example, FD of the respiratory system, cardiovascular system, skin, or eye.
- Building block C, contingent on the completion of blocks A and B, will oversee the iterative FDO development. The backbone of this block will involve aligning clinical phenomena with the ICD-11. Additional concepts will be incorporated as data and object properties as the FDO develops, with further refinement driven by future use cases and maintenance needs.
- Building block D will conduct an evaluation module to ensure the ontology’s quality and consistency.

### Collective Domain Expertise of Experienced Clinicians and Researchers Applied to Determining Scope and Analyses of Targeted Scoping Reviews

A multidisciplinary team of experienced clinicians and researchers will collaborate to determine the scope of this study and analyze the results of our targeted scoping reviews. Drawing on the expertise cultivated during the RESOLVE FP7-EU project [1], among other experiences, this team will convene biweekly throughout the study (see Table 1 for study timelines). The meetings will facilitate iterative refinement of conceptual categories and relationships within the ontology, with each stage involving a critical review of current evidence, method refinement, and a discussion of concept applicability.

**Table 1:** Overview of study timelines.

Item		Month																			
		1	2	3	4	5	6	7	8	9	10	11	12	13	14	15	16	17	18	19	20
Develop protocol		✓	✓																		
Publish protocol				✓																	
**Implement protocol**					✓	✓	✓	✓	✓	✓	✓	✓	✓	✓	✓	✓	✓	✓			
	Phase A				✓																
	Phase B					✓															
	Phase C						✓	✓	✓	✓	✓	✓	✓	✓	✓	✓	✓				
	Phase D																	✓			
Submit to BioPortal																			✓		
Publish paper																				✓	✓

To achieve consensus, we will use a modified real time Delphi method [35] when necessary. If the team cannot reach a consensus on a particular issue, we will use a 2-round voting system with straightforward digital tools. The first round entails voting on all proposed opinions, while the second round, if needed, narrows the vote to the 2 highest-ranked options from the initial round.

The scoping review will guide the identification of specific concepts within subdomains, allowing us to map FD concepts across various organs (eg, lung, liver, and kidney) and systems (eg, cardiovascular diseases). These findings will be systematically reviewed by the experts for integration into the FDO development process. We will maintain a cycle of information collection, analytical processing, and subsequent integration into the FDO framework.

### Scoping Review of Prevalent Concepts and Domain Literature

We will use a scoping review methodology to extract concepts related to the domain of FDs from the PubMed database’s peer-reviewed literature [36]. Given the breadth of our research questions and the inclusion of various study types, a scoping review provides an appropriate approach to fulfilling our objectives. The review stages will adhere to the methodological framework by Arksey and O’Malley [37], as revised by Levac et al [38]: (1) population, intervention, comparison, outcomes, and study (PICOS)-based research question formulation [39]; (2) relevant study identification through a MEDLINE and PubMed database search; (3) study screening based on inclusion and exclusion criteria; (4) data charting, including relevant paper review; and (5) collation and summary of findings. Our report will align with the Preferred Reporting Items for Systematic Reviews and Meta-Analysis–Scoping Review (PRISMA-ScR) framework [40].

#### Search Strategy

For the first phase (phase A), we will systematically search the MEDLINE and PubMed databases using the search strings *fibroproliferative disease* OR *fibrotic disease*. To map FD concepts across different organs and systems, we will pair these terms with the relevant organ and system subdomain (eg, *fibrosis* AND *lung*). Additional terms and Boolean operators may be introduced based on expert judgment and domain knowledge. Domain experts, clinicians, and researchers involved in the FDO’s development will decide on the detailed settings for these searches.

We will refine our search string through test searches, identifying appropriate synonyms, medical subject headings (MeSH) phrases, and keywords. We will also manually search the references of pertinent studies.

#### Inclusion and Exclusion Criteria

Inclusion and exclusion criteria will be established through the PICOS framework, guiding the screening process. We will include any peer-reviewed study conducted on FDs without study design-based exclusions. We will focus on full-text, peer-reviewed research published since 2019 to reflect the field’s recent developments and ensure sufficient evidence gathering. Exclusions include non-English papers, editorials, letters to the editor, commentaries, and press articles (see Multimedia Appendices 1 and 2 for eligibility screening form details).

#### Selection of Studies

The review team, comprising medical, biomedical informatics, and public health experts, will work in pairs to review papers. Each paper will be independently evaluated by 2 reviewers, with disagreements resolved through discussion (see Multimedia Appendices 1 and 2 for screening details).

#### Charting the Data

Data extraction will occur through a shared Microsoft Office (Microsoft Corp) Excel form (Multimedia Appendix 3), featuring a combination of close-ended and open-ended questions. Open-ended queries will help identify the article’s primary aim and crucial results. We will extract key details, including authorship, publication year, domain, objectives, key results, and significant FD-related concepts, facilitating the development of a domain-specific glossary and ongoing model and ontology construction.

### ICD-11: Screening Process Ensuring Globally Accepted Naming Standards and Proper Level of Granularity Across the Fibroproliferative Disease Ontology

The ICD-11 is a universally accepted tool, providing over 1.6 million terms for standardized health care information exchange [41]. It is publicly accessible, endorsed by the WHO, and used globally [42].

To ensure the interoperability of our FDO with this internationally recognized nomenclature and to maintain appropriate granularity, we will align all relevant concepts in our FDO with corresponding ICD-11 terms. This process will encompass all FDs and clinical conditions proposed for inclusion in the FDO. Therefore, we will compare the identified names of FDs with those listed in the ICD-11 classification. Each disease included in our FDO will follow ICD-11 naming conventions and be assigned a corresponding ICD-11 code.

### Formalization of Ontology Using Web Ontology Language in Protégé

The FDO will be constructed using the Semantic Web standard OWL through Protégé (Stanford University), a leading open-source ontology editor [43]. Drawing from the scoping review findings and the team’s expertise, we will incorporate and model various class entities and properties using pertinent OWL axioms, encompassing classes, object properties, data properties, and annotations. Conforming to best practices [44], relevant terms from the ICD-11 will be externally cross-referenced. The ontologies will not be imported given their distinct developmental contexts; specifically, the ICD-11 serves as an exhaustive disease reference terminology, whereas the FDO concentrates on the FD knowledge domain. Upon completion, the FDO will be uploaded to BioPortal [45], the world’s most extensive open-source repository of biomedical ontologies, to facilitate annotation and dissemination.

### Evaluation

The FDO will undergo evaluation to ensure validation and verification. The assessment will concentrate on the ontology’s consistency, clarity, and comprehensive coverage of the FD domain [46]. Consistency checks will occur both during and after the ontology’s development, with the open-source semantic reasoner Pellet (Clark and Parsia LLC) used for this purpose [47].

We will involve domain experts for qualitative evaluations. They will devise proficiency questions to examine the ontology’s validity and will provide feedback on all FDO concepts and relationships through semistructured interviews, ensuring alignment with current domain knowledge [48]. To ensure the ontology accurately mirrors intended concept meanings and to maintain its coherence, we will eliminate irrelevant or incorrect entities, supplement missing terms, and uphold consistency [49].

### Fibroproliferative Diseases Ontology Maintenance and Further Development Plan

Like any ontology, the FDO is expected to evolve continually. Realizing a comprehensive ontology for the FD domain is an iterative process, and as such, the FDO must consistently incorporate new concepts and data. It must also stay abreast of knowledge progression or alterations within the domain, ensuring accurate FDO representations. Additionally, regular maintenance by FDO biocurators is essential to rectify any irregularities or errors encountered during FDO use and development [50]. We plan to conduct FDO curation in association with the International Society of Biocuration [51].

The FDO maintenance and development strategy will incorporate 2 major cyclical steps: (1) application of FDO in FD research (use cases) and (2) annual, routine maintenance.

In step 1, subsequent to the initial FDO development, we anticipate its immediate use across multiple use cases. This includes applying FDO to analyze existing FD therapeutic strategies, causative factors, and the role of antimicrobial peptides in FD. Aside from their intrinsic value, these practical FDO applications will contribute to its maintenance and curation by highlighting inconsistencies, irregularities, and nonconformance with current knowledge.

In step 2, FDO biocurators will conduct annual updates, with web-based meetings scheduled each summer.

FDO is designed to accommodate new concepts and properties. Both its maintenance and development will involve ontology population, that is, adding new instances, and ontology enrichment, that is, adding properties to instances [52]. As the primary FDO framework is established and tested in initial use cases, we plan to gradually automate certain tasks related to steps 1 and 2. The progressive integration and reuse of concepts from existing ontologies are also planned as the FDO develops.

## Results

The FDO, developed through the methodological framework outlined above, will be made open source, thus becoming a publicly available resource for stakeholders, researchers, and clinicians. The objective of this publication is to ensure transparency and integrity during ontology creation, stimulate domain expert interest, foster acceptance, and encourage continued use once the ontology is released in the public domain. The FDO will be registered and made accessible on BioPortal. Moreover, we plan to showcase the FDO at pertinent scientific conferences through abstracts and presentations. Additionally, the finalized FDO will be disseminated as a peer-reviewed publication.

## Discussion

### Overview

The protocol we have outlined for the development of the FDO aims to provide a crucial tool for representing and structuring knowledge within the FD domain. The key benefits of representing the FD knowledge domain as an ontology lie in its structured nature, which eases the sharing and dissemination of knowledge and encourages reuse across different research teams, projects, or subspecialties [53]. For example, discoveries in oncology or hematology could spur research in pulmonary medicine. Furthermore, the FDO can facilitate uncovering new relationships or parallels between distinct research findings or identifying knowledge and research gaps [54]. The standardized, interoperable usage of terms will streamline data exchange and integration, potentially accelerating in-depth analysis of general fibroproliferative processes and disease characteristics. Such a structured approach with standardized terminology should also enhance research quality [55]. By leveraging ICD-11 and SNOMED CT terms, we ensure both standardization and consistent granularity across the ontology [56]. This approach is designed to yield a comprehensive ontology of FDs, thereby avoiding the pitfall of incomplete coverage.

However, we anticipate certain limitations with the FDO developed through this protocol, some of which are inherent to biomedical ontologies expressed in OWL description logic [56]. OWL ontologies express relationships between objects, classes, concepts, and their properties, but they do not describe processes or changes occurring over time [57]. This is a significant consideration given that FDs typically involve chronic processes such as pathologies of tissue repair, aging, and senescence. As a workaround, we may consider creating multiple instances to describe a particular FD in the ontology instead of the “one disease, one instance” setting. Despite this, such a workaround introduces other limitations like potential bias, inconsistencies, and increased complexity, which may pose challenges for efficient use and interpretation of the ontology [56].

In conclusion, while the FDO is a promising tool for the representation of the FD knowledge domain, its success hinges on regular updates to keep pace with the evolving understanding of FDs. Therefore, we have planned follow-up activities to ensure that the ontology remains current and relevant.

### Conclusions

We will create an FDO as a shared knowledge resource and conceptual framework accessible in the public domain for open usage, including by semantic reasoners and other tools. This facilitates collaboration and promotes ongoing updates and enhancements. The development of the FDO is described as an iterative process involving domain experts, scoping reviews, and the incorporation of existing standardized terminology. Once publicly available, the FDO is anticipated to stimulate research in the FD domain, driving clinical advancements, informing medical research, and influencing public health initiatives. Consequently, it will provide novel insights into the substantial current knowledge gaps in this field of study.

## Appendix

Multimedia Appendix 1 Screening phases A and B.

Multimedia Appendix 2 Eligibility screening form on the basis of full-text screening in phases A and B.

Multimedia Appendix 3 Data extraction: characterization form on the basis of full-text analysis—phases A and B.

## Abbreviations

ECM: extracellular matrix
EMBL-EBI: European Molecular Biology Laboratory-European Bioinformatics Institute
EMT: epithelial-mesenchymal transition
FD: fibroproliferative or fibrotic diseases
FDO: fibroproliferative or fibrotic disease ontology
FP7-EU: Seventh Framework Program of the European Union
ICD-11: International Classification of Diseases 11th Revision
IPF: idiopathic pulmonary fibrosis
MeSH: medical subject headings
OWL: Web Ontology Language
PICOS: Population, Intervention, Comparison, Outcomes, and Study
PRISMA-ScR: Preferred Reporting Items for Systematic Reviews and Meta-Analysis—Scoping Review
RESOLVE: Resolve Chronic Inflammation and Achieve Healthy Aging by Understanding Non-regenerative Repair
SNOMED CT: Systematized Nomenclature of Medicine–Clinical Terms
UMLS: Unified Medical Language System
WHO: World Health Organization

## Data Availability

All data generated or analyzed during this study are included in this published article and its supplementary information files.
