# Supplementary figures and images for: Comprehensive Ontology of Fibroproliferative Diseases: Protocol for a Semantic Technology Study
Source: JMIR Res Protoc. 2023 Aug 11;12:e48645. doi: 10.2196/48645 (PMC10457705; doi:10.2196/48645)

**Multimedia Appendix 1.** Screening: Phases A and B.

**
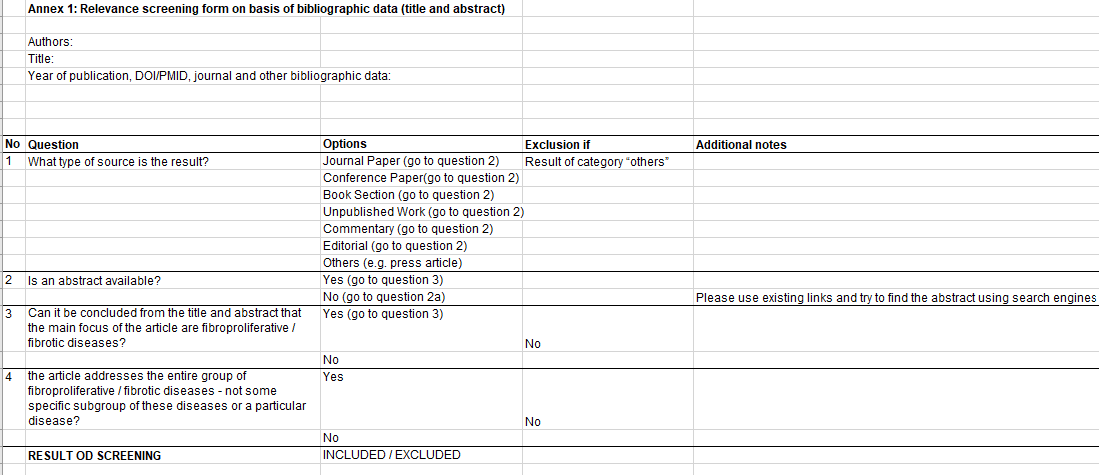
**

Supplement: Multimedia Appendix 1 [file resprot_v12i1e48645_app1.docx]
