# Supplementary material for: Comprehensive Ontology of Fibroproliferative Diseases: Protocol for a Semantic Technology Study
Source: JMIR Res Protoc. 2023 Aug 11;12:e48645. doi: 10.2196/48645 (PMC10457705; doi:10.2196/48645)

**Multimedia Appendix 3.** Data extraction: characterization form on the basis of full-text analysis—Phases A and B.


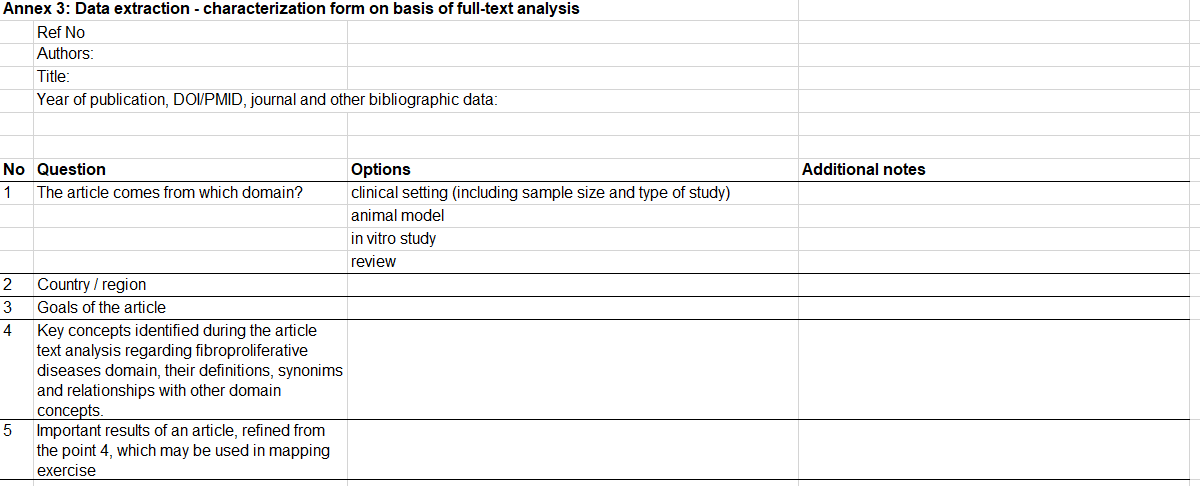

Supplement: Multimedia Appendix 3 [file resprot_v12i1e48645_app3.docx]
